# Circ‐Bnc2 alleviates neuroinflammation in LPS‐stimulated microglial cells to inhibit neuron cell apoptosis through regulating miR‐497a‐5p/HECTD1 axis

**DOI:** 10.1002/brb3.2935

**Published:** 2023-03-24

**Authors:** Yanfang Chen, Peng Cao

**Affiliations:** ^1^ The First Affiliated Hospital, Department of Neurology, Hengyang Medical School University of South China Hengyang China; ^2^ The First Affiliated Hospital, Department of Hepatopancreatobiliary Surgery, Hengyang Medical School University of South China Hengyang China

**Keywords:** circ‐Bnc2, depression, HECTD1, mir‐497a‐5p, neuroinflammation

## Abstract

**Background:**

Neuroinflammation caused by microglia cells activation and the apoptosis of neuron cells are associated with the occurrence of depression. Circ‐Bnc2 has been shown to be significantly downregulated in depression mice, but its role in the progression of depression remains unclear.

**Methods:**

Lipopolysaccharide (LPS) was used to treat BV2 microglial cells to induce neuroinflammation. The expression of circ‐Bnc2, microRNA (miR)‐497a‐5p, and HECT domain E3 ubiquitin protein ligase 1 (HECTD1) was measured by quantitative real‐time PCR. The protein levels of neuroinflammation markers, apoptosis markers, and HECTD1 were determined by western blot analysis. ELISA assay was used to examine the concentrations of inflammatory factors. After HT22 cells were cultured with the conditioned medium of LPS‐induced BV2 cells, the proliferation and apoptosis of HT22 cells were assessed by cell counting kit 8 assay, EdU assay, and flow cytometry. In addition, the interaction between miR‐497a‐5p and circ‐Bnc2 or HECTD1 was confirmed by dual‐luciferase reporter assay, RIP assay, and RNA pull‐down assay.

**Results:**

Our data showed that circ‐Bnc2 was lowly expressed in LPS‐induced BV2 cells. Function experiments suggested that circ‐Bnc2 could inhibit LPS‐induced neuroinflammation in BV2 cells to repress HT22 cell apoptosis and promote proliferation. Circ‐Bnc2 could sponge miR‐497a‐5p, and the neuroprotective function of circ‐Bnc2 could be reversed by miR‐497a‐5p overexpression. Additionally, miR‐497a‐5p could target HECTD1. miR‐497a‐5p inhibitor could alleviate LPS‐induced neuroinflammation in BV2 cells and reduce HT22 cell apoptosis, which also could be reversed by HECTD1 knockdown. Moreover, circ‐Bnc2 had a positive regulation on HECTD1 expression by sponging miR‐497a‐5p.

**Conclusion:**

In summary, our results confirmed that circ‐Bnc2 could inhibit neuroinflammation and neuron cell apoptosis by regulating miR‐497a‐5p/HECTD1 axis, suggesting that circ‐Bnc2 might be a potential target for depression treatment.

## Graphical abstract

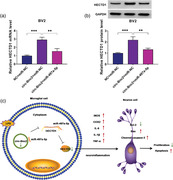

## 1 INTRODUCTION

Depression is a common mental illness, and its high incidence and disability rate have brought a heavy burden to the society and patients’ families (Casey, 2017; Smith, 2014). In recent years, with the discovery of neurobiology, the role of neuroinflammation in the occurrence of depression has received more and more attention (Carlessi et al., 2021; Troubat et al., 2021). Chronic stress is the main trigger factor of depression, which can lead to a large increase of pro‐inflammatory cytokines in the brain, and then contribute to central neuroinflammation (Seo et al., 2017). Microglia are the main effector cells mediating neuroinflammation, and their overactivation can produce a large number of pro‐inflammatory cytokines, resulting in neuronal apoptosis (Woodburn et al., 2021). The hippocampus is one of the limbic system structures related to emotional diseases and also an important brain region mediating stress response (Snyder et al., 2011). In recent years, more and more attention has been paid to the relationship between adult hippocampal neuronal injury and depression. Studies have shown that patients with depression have hippocampal injury accompanied by increased apoptosis of hippocampal neuronal (Peng et al., 2020). Many studies have revealed that microglia activation caused neuroinflammation and neuron cell apoptosis is an important pathological basis for depression (Brites & Fernandes, 2015; Xu et al., 2020). Elucidating the molecular mechanisms affecting neuroinflammation is expected to provide a theoretical basis for depression treatment.

Lipopolysaccharide (LPS) is a component of the cell wall of Gram‐negative bacteria, which can induce severe inflammation response in the peripheral and brain (Ramires et al., 2021). As a cytokine inducer, LPS can induce behaviors related to anxiety and depression, and lead to central nervous system dysfunction (Nava Catorce & Gevorkian, 2016). LPS has been used to induce a variety of inflammatory injury models, including neuroinflammation model in depression (Zhang et al., 2016).

Circular RNA (circRNA) is a noncoding RNA molecule, and their unique circular structure makes them highly stable and able to escape the digestion of exonuclease (Fang et al., 2020; Kristensen et al., 2019). In terms of mechanism, circRNA has been proved to be involved in posttranscriptional regulation of genes, which can act as a microRNA (miRNA) sponge to indirectly regulate target gene expression (Dori & Bicciato, 2019; Panda, 2018). Studies have shown that circRNA may be involved in the pathogenesis of depression, and there is a certain relationship with the symptoms and severity of depression (Gan et al., 2021; Li et al., 2020). For example, Bu et al. (2021) suggested that circ_0126218 was significantly upregulated in female depression patients, which could be used as a diagnostic biomarker for depression. Moreover, circDYM was downregulated in LPS‐treated mice and hippocampal neuron cells, and its overexpression could attenuate depressive‐like behavior in mice and repress microglial activation by upregulating HECT domain E3 ubiquitin protein ligase 1 (HECTD1) through sponging miR‐9 (Zhang et al., 2020).

In a previous study, mmu_circ_0001223 (derived from Bnc2 gene, also called circ‐Bnc2) was found to be significantly downregulated in a chronic unpredictable mild stress mice model, and its overexpression could promote the protein expression of CREB1 and BDNF to alleviate depression progression (Zhang et al., 2018). Therefore, circ‐Bnc2 might be a vital circRNA regulating depression progression. Here, we explored the function of circ‐Bnc2 in LPS‐induced microglial cell neuroinflammation and neuron cell apoptosis to reveal its role in depression progression. Through the hypothesis of circRNA/miRNA/mRNA axis, we further revealed the potential molecular mechanism of circ‐Bnc2 in regulating depression progression.

## 2 MATERIALS AND METHODS

### 2.1 Cell culture and LPS treatment

Mouse microglia cells (BV2) and hippocampal neuron cells (HT22) were purchased from Procell (Wuhan, China). BV2 cells were cultured in MEM medium (Sigma–Aldrich, St. Louis, MO, USA) and HT22 cells were grown at DMEM medium (Sigma–Aldrich) at 37°C with 5% CO_2_. The medium contained 10% FBS (Gibco, Grand Island, NY, USA) and 1% Penicillin–Streptomycin Solution (Procell). BV2 cells were treated with different concentrations of LPS (Sigma–Aldrich) for 24 h or 1 μg/mL LPS for different times.

### 2.2 Cell transfection

BV2 cells were seeded into six‐well plates and cultured until the cells reached 60% confluences. The circ‐Bnc2 overexpression vector, miR‐497a‐5p mimic or inhibitor, HECTD1 small interference RNA (si‐HECTD1), and their negative controls were synthesized by RiboBio (Guangzhou, China), and they were transfected into BV2 cells using Lipofectamine 3000 (Invitrogen, Carlsbad, CA, USA). After transfection for 24 h, BV2 cells were treated with 1 μg/mL LPS for 24 h.

### 2.3 Quantitative real‐time PCR

Total RNAs were obtained from BV2 cells using RNAiso Plus (TaKaRa, Dalian, China), and then reverse‐transcribed into cDNA using cDNA synthesis kit (Bio‐Rad, Hercules, CA, USA). SYBR premix Ex Taq II Kit (TaKaRa) was used to perform PCR reaction in PCR system (Bio‐Rad). Data were normalized by GAPDH or U6 and calculated by 2^−ΔΔCt^ method. Primer sequences were listed in Table 1.

**TABLE 1:** Primers sequences used for qRT‐PCR

Name		Primers for PCR (5′−3′)
mmu_circ_0001223 (circ‐Bnc2)	Forward	TCGTCGAAGCAGAGACAGGA
	Reverse	ATTTGGTTTCCCCAAACCGC
HECTD1	Forward	TGACAATGTGGATCGCTGTTT
	Reverse	TACTATCCTCCGGGTGCATTC
miR‐497a‐5p	Forward	GGTATGACAGCAGCACACTGT
	Reverse	CTCAACTGGTGTCGTGGAGTC
GAPDH	Forward	GGAGAGTGTTTCCTCGTCCC
	Reverse	ACTGTGCCGTTGAATTTGCC
U6	Forward	CTCGCTTCGGCAGCACATATACT
	Reverse	ACGCTTCACGAATTTGCGTGTC

### 2.4 Identification of circ‐Bnc2

In order to verify the circular characteristic of circ‐Bnc2, we performed divergent primers amplification assay, RNase R assay, and subcellular localization assay. The transcript of circ‐Bnc2 was amplified from genomic DNA (gDNA) and cDNA of BV2 cells using divergent primers and convergent primers, respectively, and the PCR products were separated by agarose gel electrophoresis. In addition, the RNA (1 μg) extracted from BV2 cells was treated with 3 U/μg RNase R (Geneseed, Guangzhou, China) for 15 min, and then the expression of circ‐Bnc2 and GAPDH was analyzed by quantitative real‐time PCR (qRT‐PCR) to determine the resistance of circ‐Bnc2 to RNase R. At the same time, the PARIS Kit (Invitrogen) was used to separate the cytoplasmic and nuclear RNA from BV2 cells, and then the expression of circ‐Bnc2, U6, and GAPDH was tested by qRT‐PCR to determine the location of circ‐Bnc2 in the cytoplasm and nucleus.

### 2.5 Western blot analysis

Total proteins were extracted by RIPA lysis buffer (Beyotime, Shanghai, China) and quantified by BCA Kit (Beyotime). Equal amount of protein (30 μg) was separated by 10% SDS‐PAGE and transferred onto PVDF membranes. The membranes were blocked with 5% nonfat milk, and then hatched with the primary antibody including anti‐iNOS (1:1000, ab178945; Abcam), anti‐COX2 (1:1000, ab179800; Abcam), anti‐Bcl‐2 (1:2000, ab182858; Abcam), anti‐Bax (1:2000, ab182733; Abcam), anti‐cleaved caspase‐3 (1:5000, ab214430; Abcam), anti‐HECTD1 (1:1000, 20605‐1‐AP; Proteintech, Rosemont, IL, USA), and anti‐GAPDH (1:2500, ab9485; Abcam) at 4°C overnight. Then, the membranes were treated with secondary antibodies (1:50,000, ab205718; Abcam) for 1 h, and the protein blots were visualized by ECL luminescence reagent (Sangon, Shanghai, China). Grayscale analysis was carried out with Image lab software.

### 2.6 ELISA assay

After transfection or treatment, the concentrations of inflammatory cytokines IL‐6, IL‐1β, and TNF‐α in the culture supernatants of BV2 cells were detected by mouse IL‐6, IL‐1β, and TNF‐α ELISA Kits (Shanghai Enzyme‐linked Biotechnology Co., Ltd, Shanghai, China) according to the manufacturer's instructions.

### 2.7 Cell counting kit 8 assay

After transfection and treatment, the cell culture supernatant of BV2 cells was collected and then co‐cultured with HT22 cells for 12 h. After that, HT22 cells were inoculated into 96‐well plates (1 × 10^4^ cells/well) and cultured for 48 h. Then, cell counting kit 8 (CCK8) solution (10 μL) was added into cells and incubated for 4 h. Cell viability was measured at 450 nm using a microplate reader.

### 2.8 EdU staining

EdU Cell proliferation Detection Kit (RiboBio) was used to measure cell proliferation. In brief, HT22 cells were incubated with the cell culture supernatant of BV2 cells for 12 h, and then were re‐plated onto 24‐well plates (4 × 10^4^ cells/well). After 24 h, HT22 cells were labeled with EdU solution and stained with DAPI according to the kit instructions. Fluorescence images were acquired by fluorescence microscopy and the EdU positive (EdU^+^) cell rate was counted using Image J software.

### 2.9 Flow cytometry

After BV2 cells were co‐cultured with the cell culture supernatant, HT22 cells were harvested (3 × 10^5^ cells) and then re‐suspended in 100 μL Annexin‐binding buffer (Vazyme, Nanjing, China). Afterward, the cells were incubated with Annexin‐V‐FITC and PI (Vazyme) for 10 min, and the apoptotic cell rate was analyzed by a flow cytometer with CellQuest software.

### 2.10 Dual‐luciferase reporter assay

The sequences of circ‐Bnc2 or HECTD1 3′UTR containing the predicted sites or mutant sites with miR‐497a‐5p were inserted into the pGL3 control vector to generate the WT/MUT‐circ‐Bnc2 vectors and WT/MUT‐HECTD1 3′UTR vectors. BV2 cells were co‐transfected with the above reporter vectors and miR‐497a‐5p mimic or inhibitor. Relative luciferase activity was measured with Dual‐Luciferase Reporter Assay Kit (Vazyme).

### 2.11 RIP assay

RIP Kit (Millipore, Billerica, MA, USA) was used for RIP assay. BV2 cells were lysed using RIP lysis buffer, and the cell supernatants were incubated with magnetic beads coated with anti‐Ago2 or anti‐IgG. After incubation for 4 h, the enrichments of circ‐Bnc2, miR‐497a‐5p, and HECTD1 were examined by qRT‐PCR.

### 2.12 RNA pull‐down assay

The biotin‐labeled probes were synthesized by Sangon, including wild‐type or mutant‐type miR‐497a‐5p probe (WT/MUT‐bio‐miR‐497a‐5p) and its negative control probe (bio‐NC). The probes were transfected into BV2 cells. After 48 h, cell lysates were hatched with streptavidin‐labeled magnetic beads. The enrichments of circ‐Bnc2 and HECTD1 were determined by qRT‐PCR.

### 2.13 Statistical analysis

GraphPad Prism v7.0 was used to analyze the experimental data. Data were expressed as means ± SD. Statistical analysis was performed by Student's *t*‐test or one‐way ANOVA. A *p*‐value < .05 was considered statistically significant.

## 3 RESULTS

### 3.1 Circ‐Bnc2 expression was downregulated in LPS‐induced BV2 cells

**FIGURE 1:**
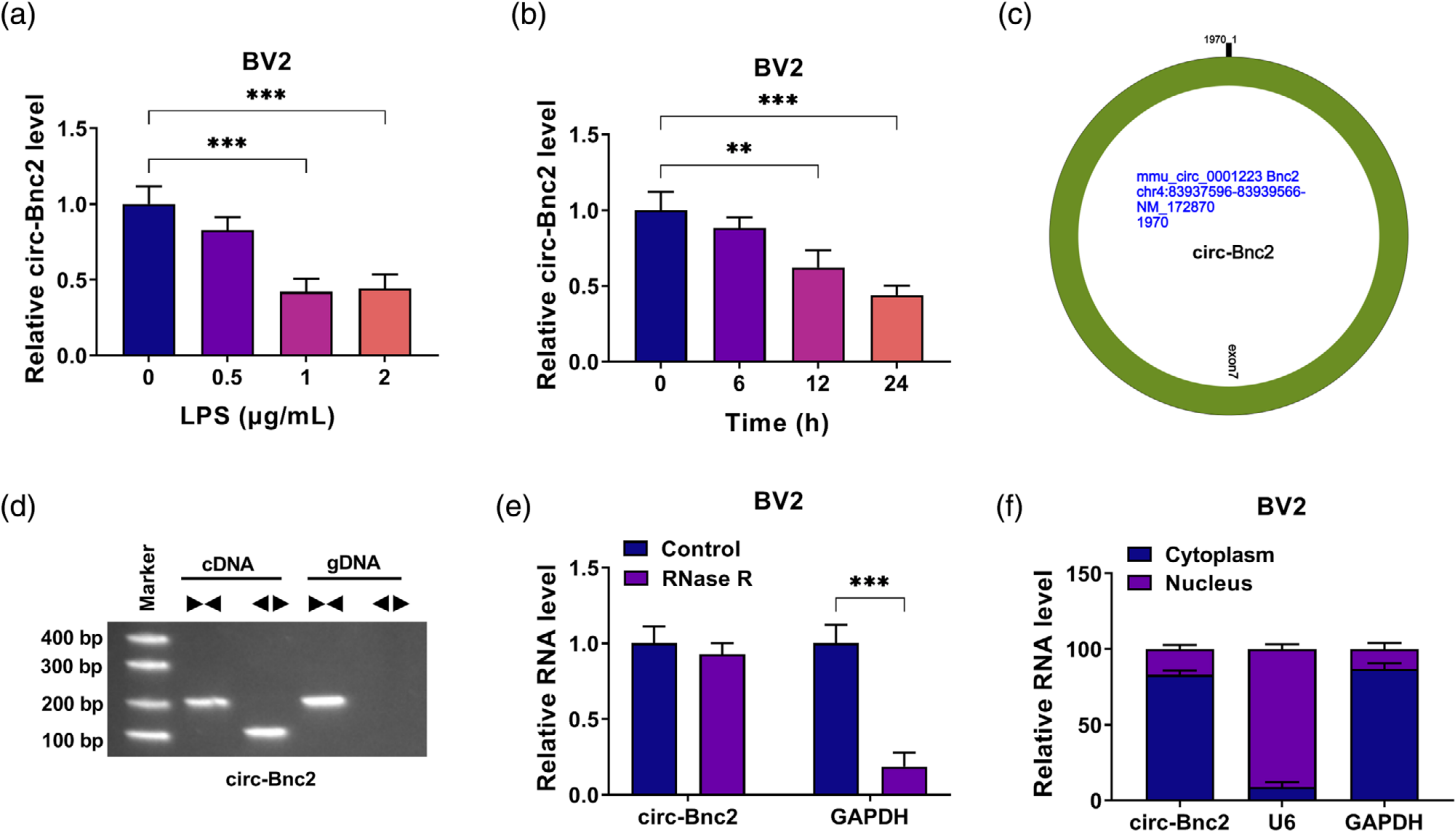
Circ‐Bnc2 expression was downregulated in LPS‐induced BV2 cells. (A) qRT‐PCR was used to detect the expression of circ‐Bnc2 in BV2 cells treated with different concentrations of LPS for 24 h. (B) qRT‐PCR was performed to measure the expression of circ‐Bnc2 in BV2 cells treated with 1 μg/mL LPS for different times. (C) The basic information of circ‐Bnc2 was shown. (D) The cDNA and gDNA of circ‐Bnc2 were amplified with divergent primers and convergent primers using qRT‐PCR, and the amplified products were analyzed by agarose gel electrophoresis. (E) RNase R assay was used to confirm the resistance of circ‐Bnc2 on RNase R. (F) Subcellular localization analysis was used to confirm the distribution of circ‐Bnc2 in the cytoplasm and nucleus of BV2 cells. **p < .01; ***p < .001.

In different concentrations of LPS treatment for 24 h, we found that circ‐Bnc2 was significantly downregulated in BV2 cells treated with 1 and 2 μg/mL LPS (Figure 1a). In addition, the expression of circ‐Bnc2 was also significantly downregulated in BV2 cells after treatment with 1 μg/mL LPS for 12 and 24 h (Figure 1b). Therefore, 1‐μg/mL LPS treatment was selected for 24 h in subsequent experiments. Using the circPrimer1.6 software, we confirm that circ‐Bnc2 is located at chr4 and is formed by the back‐splicing of Bnc2 gene (Figure 1c). Subsequently, we also used different primers to amplify circ‐Bnc2 in BV2 cells, and found that divergent primers could be amplified in cDNA, but not in gDNA (Figure 1d). RNase R assay results showed that circ‐Bnc2 could resist the digestion of RNase R compared to linear RNA GAPDH (Figure 1e). Subcellular localization analysis showed that circ‐Bnc2 was mainly existed in the cytoplasm of BV2 cells (Figure 1f). The above results suggested that circ‐Bnc2 was indeed a circRNA and might be mainly involved in posttranscriptional regulation.

### 3.2 Circ‐Bnc2 repressed HT22 cell apoptosis via inhibiting LPS‐induced neuroinflammation in BV2 cells

**FIGURE 2:**
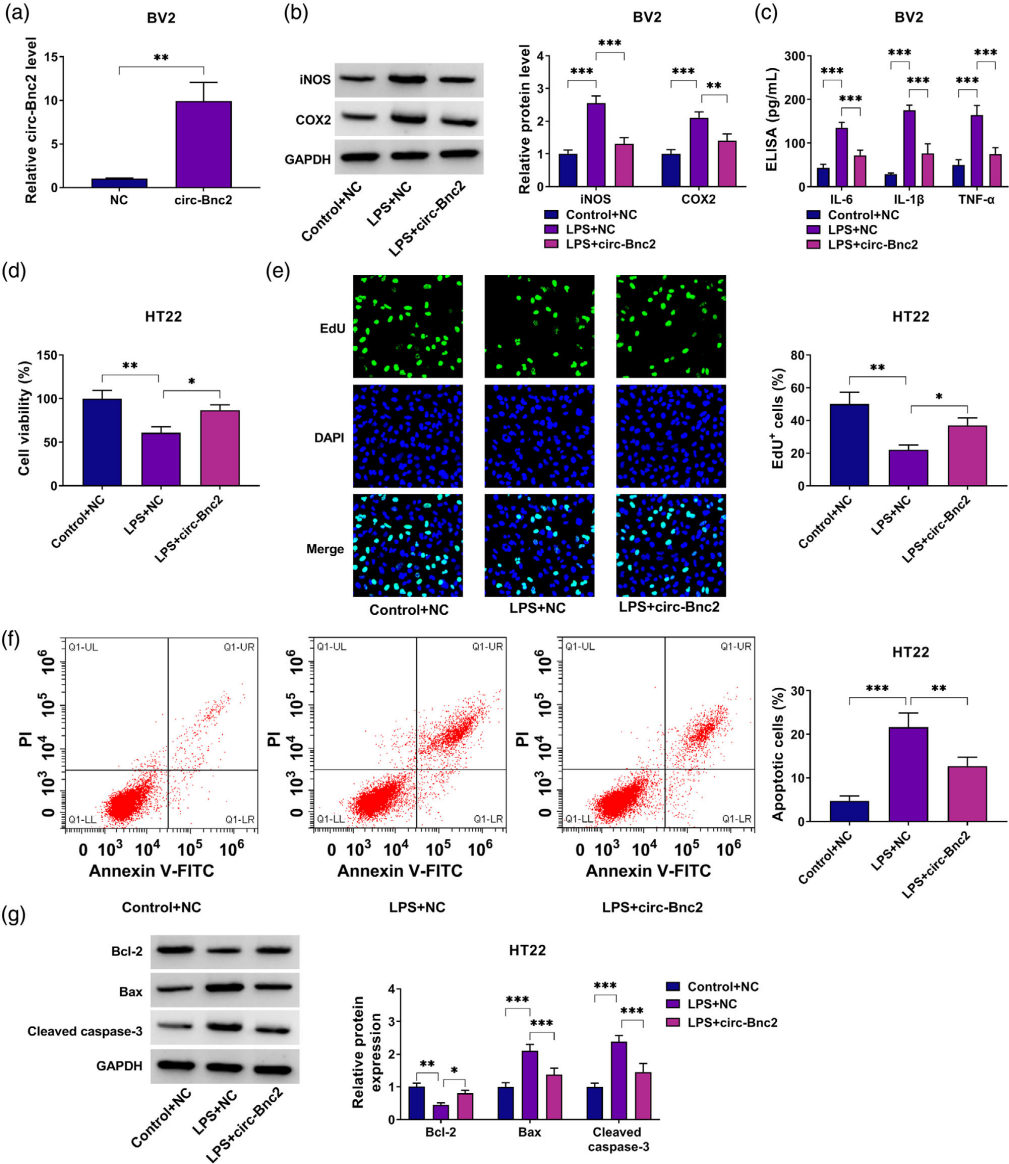
Circ‐Bnc2 repressed HT22 cell apoptosis via inhibiting LPS‐induced neuroinflammation in BV2 cells. (A) The expression of circ‐Bnc2 was measured by qRT‐PCR in BV2 cells transfected with circ‐Bnc2 overexpression vector or negative control (NC) vector. (B, C) BV2 cells were transfected with circ‐Bnc2 overexpression vector or NC vector and then treated with or without LPS. (B) The protein levels of iNOS and COX2 were determined by western blot analysis. (C) The concentrations of IL‐6, IL‐1β, and TNF‐α were analyzed by ELISA assay. (D–G) The cell culture supernatant from LPS‐stimulated BV2 cells transfected with circ‐Bnc2 overexpression vector or NC vector was co‐cultured with HT22 cells for 12 h. Cell viability, EdU^+^ cells, and apoptotic cells were determined by CCK8 assay (D), EdU assay (E), and flow cytometry (F). (G) The protein levels of Bcl‐2, Bax, and cleaved caspase‐3 were tested by western blot analysis. *p < .05; **p < .01; ***p < .001.

To assess the role of circ‐Bnc2 in neuroinflammation, we overexpressed circ‐Bnc2 in BV2 cells using circ‐Bnc2 overexpression vector (Figure 2a). After BV2 cells were treated with LPS, we found that the protein levels of iNOS and COX2 were markedly enhanced, while overexpressed circ‐Bnc2 could inhibit the protein levels of iNOS and COX2 in LPS‐treated BV2 cells (Figure 2b). Also, LPS promoted the concentrations of IL‐6, IL‐1β, and TNF‐α in BV2 cells, and circ‐Bnc2 overexpression could reduce the concentrations of inflammation factors in LPS‐induced BV2 cells (Figure 2c). The above data showed that circ‐Bnc2 could inhibit LPS‐induced BV2 cells neuroinflammation. After that, the cell culture supernatant from LPS‐stimulated BV2 cells transfected with circ‐Bnc2 overexpression vector was co‐cultured with HT22 cells for 12 h to assess the role of circ‐Bnc2 on the proliferation and apoptosis of neuron cells. Our data showed that LPS‐induced BV2 cell medium could suppress the viability and EdU^+^ cells of HT22 cells, while circ‐Bnc2 overexpression could reverse these effects (Figure 2d,e). Also, LPS‐derived BV2 cell medium enhanced the apoptotic cell rate of HT22 cells, and circ‐Bnc2 overexpression also could abolish this effect (Figure 2f). In addition, we found that Bcl‐2 protein expression was decreased, while Bax and cleaved caspase‐3 protein expression was increased in HT22 cells cultured with LPS‐derived BV2 cell medium. However, overexpressed circ‐Bnc2 also enhanced the Bcl‐2 protein expression and reduced the Bax and cleaved caspase‐3 protein expression (Figure 2g). These data confirmed that circ‐Bnc2 had a neuroprotective function.

### 3.3 Circ‐Bnc2 directly interacted with miR‐497a‐5p

**FIGURE 3:**
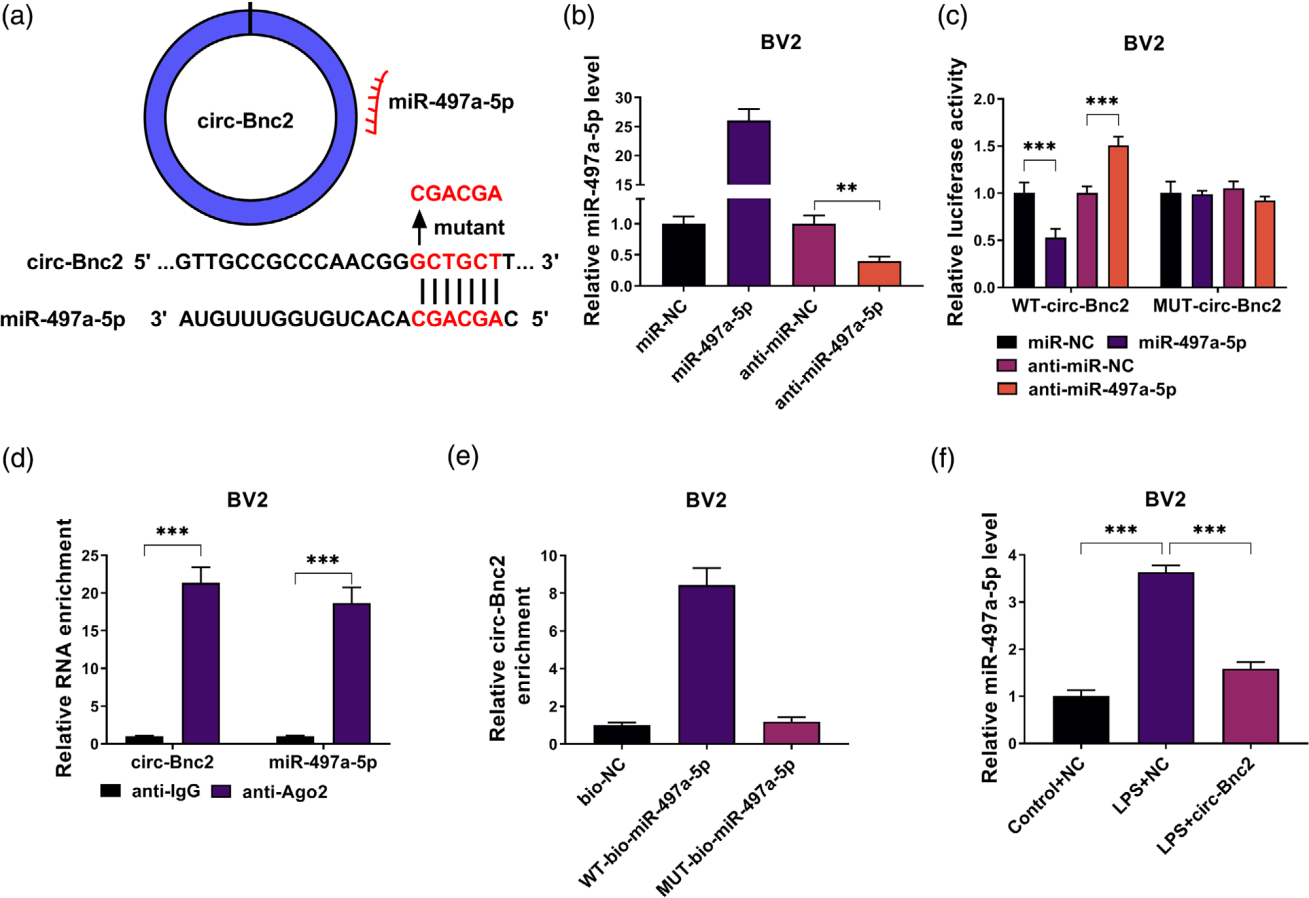
Circ‐Bnc2 directly interacted with miR‐497a‐5p. (A) The binding sites between circ‐Bnc2 and miR‐497a‐5p were exhibited. (B) MiR‐497a‐5p expression was detected by qRT‐PCR to explore whether the transfection of miR‐497a‐5p mimic and inhibitor was successful. Dual‐luciferase reporter assay (C), RIP assay (D), and RNA pull‐down assay (E) were used to confirm the interaction between circ‐Bnc2 and miR‐497a‐5p. (F) The miR‐497a‐5p expression was measured by qRT‐PCR in LPS‐induced BV2 cells transfected with NC or circ‐Bnc2. **p < .01; ***p < .001.

To search for the targeted miRNA for circ‐Bnc2, the circAtlas 2.0 software was used. As a result, miR‐497a‐5p was found to have binding sites with circ‐Bnc2 (Figure 3a). Then, miR‐497a‐5p mimic and inhibitor were constructed, and it was confirmed that miR‐497a‐5p expression was markedly enhanced and reduced by miR‐497a‐5p mimic and inhibitor in BV2 cells, respectively (Figure 3b). Using the dual‐luciferase reporter assay, we discovered that the luciferase activity of WT‐circ‐Bnc2 could be inhibited by miR‐497a‐5p mimic and increased by miR‐497a‐5p inhibitor, but the luciferase activity of MUT‐circ‐Bnc2 was not affected by miR‐497a‐5p mimic or inhibitor (Figure 3c). RIP assay was further performed to assess the interaction between miRNA and circRNA or target genes by detecting the enrichment of genes in the immunopurification of Ago2. The results indicated that the enrichment of circ‐Bnc2 and miR‐497a‐5p was significantly increased in anti‐Ago2 (Figure 3d). In RNA pull‐down assay, we found that circ‐Bnc2 could be enriched in the WT‐bio‐miR‐497a‐5p probe rather than the MUT‐bio‐miR‐497a‐5p probe (Figure 3e). These data confirmed the interaction between miR‐497a‐5p and circ‐Bnc2. In LPS‐induced BV2 cells, we discovered that miR‐497a‐5p was highly expressed compared to the control cells, and its expression was markedly reduced by circ‐Bnc2 overexpression (Figure 3f).

### 3.4 Circ‐Bnc2 exerted neuroprotective function by sponging miR‐497a‐5p

**FIGURE 4:**
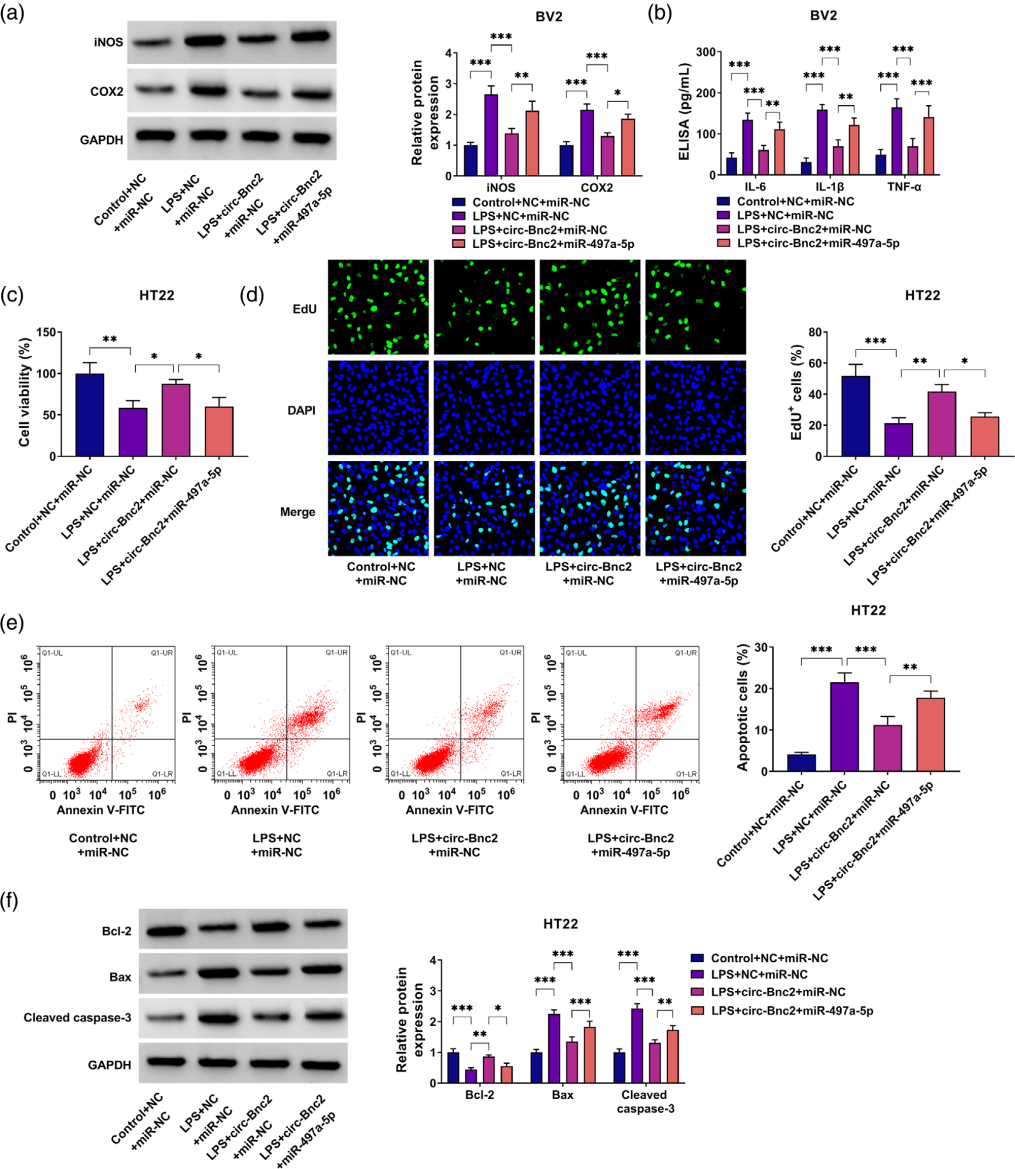
Circ‐Bnc2 exerted neuroprotective function by sponging miR‐497a‐5p. (A, B) BV2 cells were transfected with NC + miR‐NC, circ‐Bnc2 + miR‐NC, or circ‐Bnc2 + miR‐497a‐5p followed by being treated with or without LPS. (A) Western blot (WB) analysis was performed to examine the protein levels of iNOS and COX2. (B) The concentrations of IL‐6, IL‐1β, and TNF‐α were detected by ELISA assay. (C–F) The cell culture supernatant from LPS‐stimulated BV2 cells transfected with NC + miR‐NC, circ‐Bnc2 + miR‐NC, or circ‐Bnc2 + miR‐497a‐5p was co‐cultured with HT22 cells for 12 h. Cell viability, EdU^+^ cells, and apoptotic cells were determined by CCK8 assay (C), EdU assay (D), and flow cytometry (E). (F) WB analysis was used to examine the protein levels of Bcl‐2, Bax, and cleaved caspase‐3. *p < .05; **p < .01; ***p < .001.

To explore whether circ‐Bnc2 regulated LPS‐induced neuroinflammation and neuron cells apoptosis by sponging miR‐497a‐5p, we performed the rescue experiments. First, BV2 cells were transfected with circ‐Bnc2 overexpression vector and miR‐497a‐5p mimic followed by treated with LPS. Our data suggested that the inhibition effect of circ‐Bnc2 on the protein levels of iNOS and COX2, as well as the concentrations of IL‐6, IL‐1β and TNF‐α in LPS‐induced BV2 cells could be reversed by miR‐497a‐5p overexpression (Figure 4a,b). Then, the cell culture supernatant from transfected and treated BV2 cells was co‐cultured with HT22 cells. The detection results of HT22 cells proliferation showed that the promotion effect of circ‐Bnc2 on the viability and the EdU^+^ cells could be abolished by miR‐497a‐5p mimic (Figure 4c,d). Moreover, miR‐497a‐5p overexpression also reversed the suppressive effect of circ‐Bnc2 on the apoptosis of HT22 cells, showing that the apoptotic cells was enhanced, Bcl‐2 protein expression was decreased, and the Bax and cleaved caspase‐3 protein expression was increased in HT22 cells cultured with the cell culture supernatant from LPS‐induced BV2 cells transfected with circ‐Bnc2 overexpression vector and miR‐497a‐5p mimic (Figure 4e,f). All data showed that circ‐Bnc2 inhibited the neuroinflammation of microglia cells to reduce neuron cells apoptosis by sponging miR‐497a‐5p.

### 3.5 miR‐497a‐5p targeted HECTD1

**FIGURE 5:**
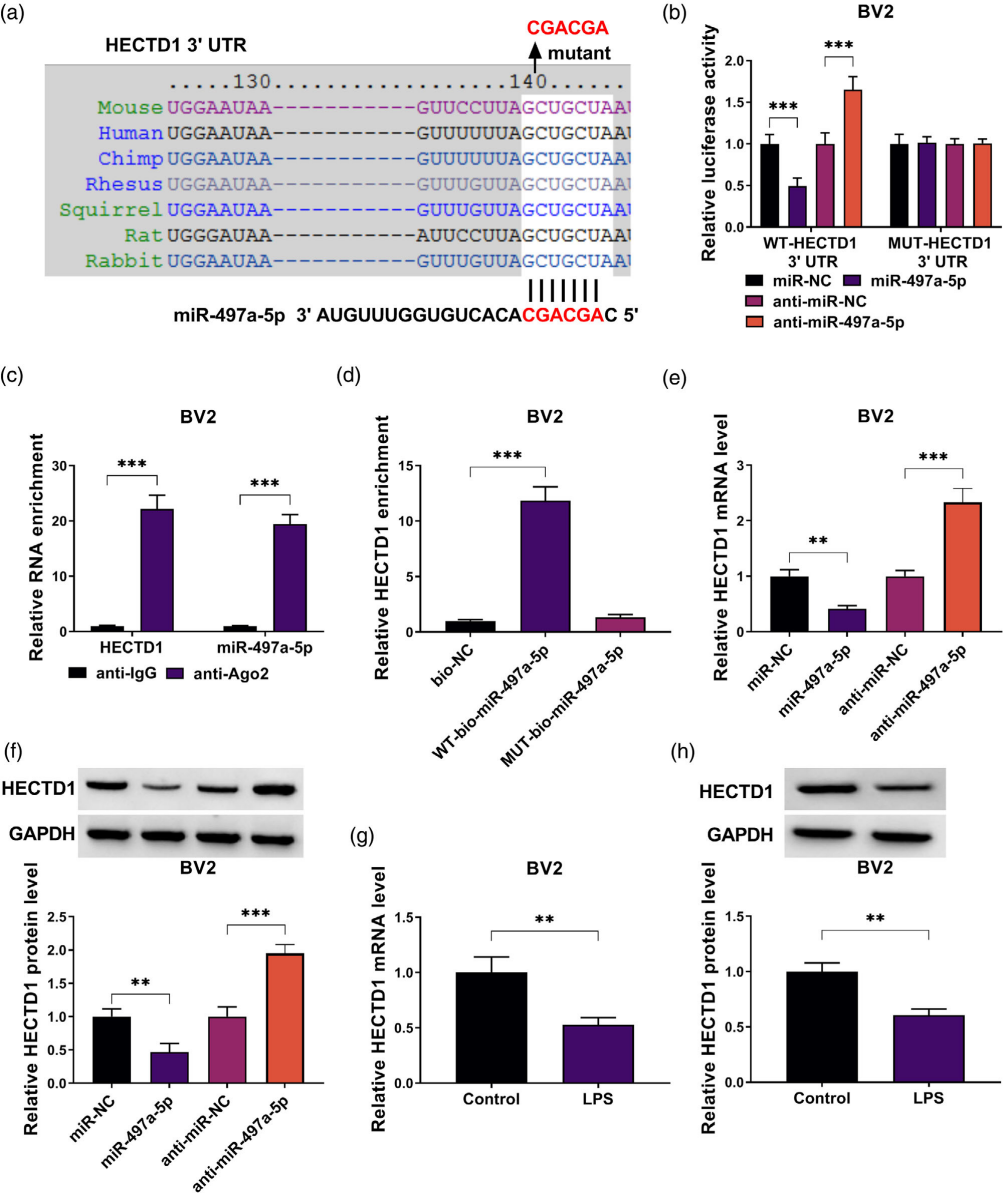
MiR‐497a‐5p targeted HECTD1. (A) The binding sites between miR‐497a‐5p and HECTD1 3′UTR were shown. The interaction between miR‐497a‐5p and HECTD1 was confirmed by dual‐luciferase reporter assay (B), RIP assay (C), and RNA pull‐down assay (D). (E, F) The mRNA and protein expression of HECTD1 was detected by qRT‐PCR and western blot (WB) analysis in BV2 cells transfected with miR‐497a‐5p mimic or inhibitor. (G, H) The mRNA and protein expression of HECTD1 was measured by qRT‐PCR and WB analysis in BV2 cells treated with or without LPS. **p < .01; ***p < .001.

Subsequently, we used TargetScan software to predict the downstream target of miR‐497a‐5p, and found that there had a targeted binding site between HECTD1 3′UTR and miR‐497a‐5p (Figure 5a). The results of dual‐luciferase reporter assay suggested that the luciferase activity of WT‐HECTD1 3′UTR vector rather than MUT‐HECTD1 3′UTR vector could be reduced by miR‐497a‐5p mimic and enhanced by miR‐497a‐5p inhibitor (Figure 5b). Besides, RIP assay results showed that HECTD1 and miR‐497a‐5p were remarkably enriched in anti‐Ago2 (Figure 5c), and RNA pull‐down assay results indicated that the enrichment of HECTD1 was significantly enhanced in the WT‐bio‐miR‐497a‐5p probe (Figure 5d). These data confirmed that HECTD1 was a target of miR‐497a‐5p. To further confirm the regulation of miR‐497a‐5p on HECTD1 expression, we measured the mRNA and protein expression of HECTD1 in BV2 cells transfected with miR‐497a‐5p mimic or inhibitor. Our data exhibited that HECTD1 expression could be decreased by miR‐497a‐5p overexpression and increased by miR‐497a‐5p inhibition at the mRNA level and protein level (Figure 5e,f). In addition, we found that the mRNA and protein levels of HECTD1 were significantly downregulated in LPS‐induced BV2 cells (Figure 5g,h).

### 3.6 miR‐497a‐5p targeted HECTD1 to regulate the neuroinflammation of LPS‐induced BV2 cells and the apoptosis of HT22 cells

**FIGURE 6:**
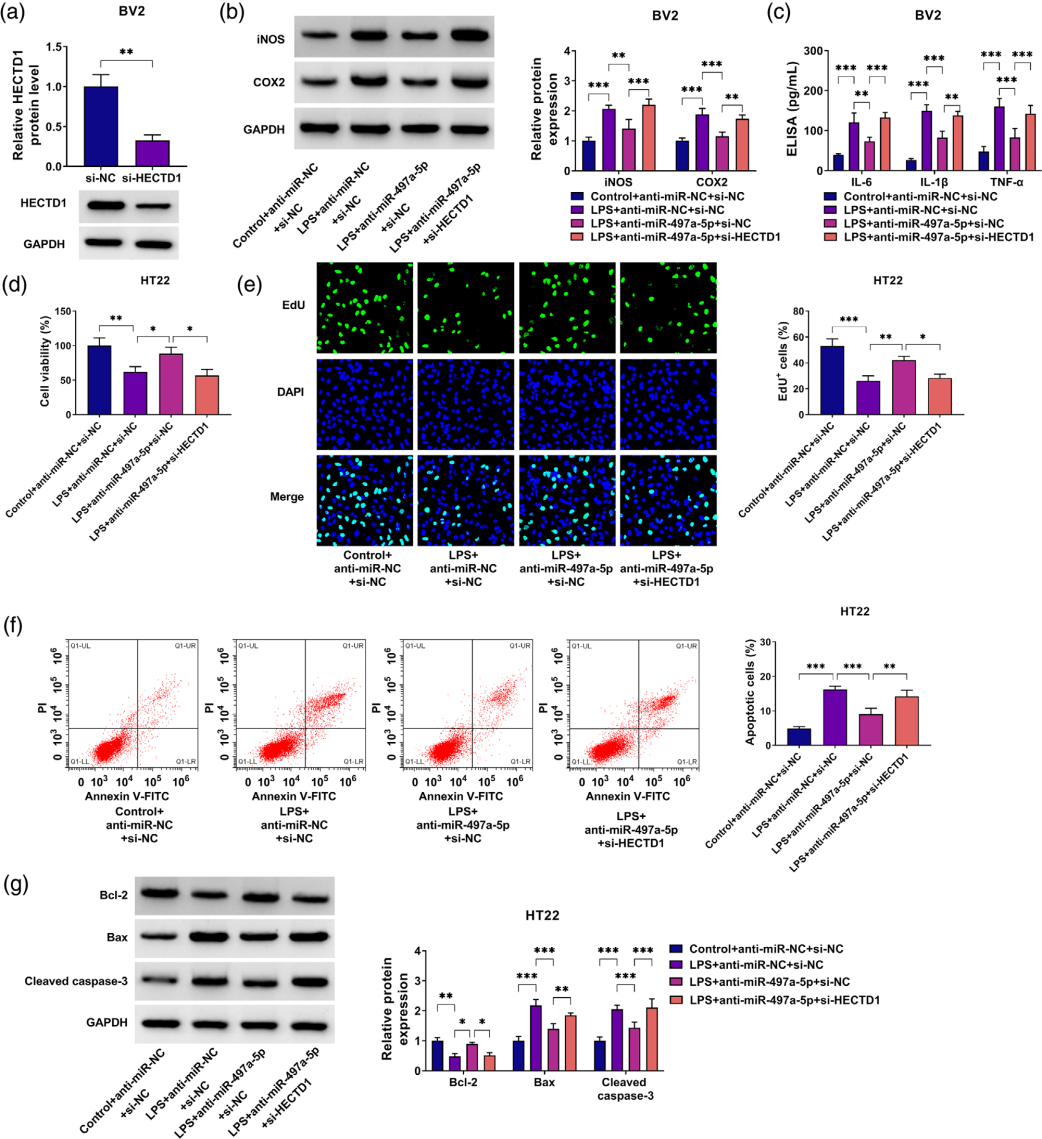
MiR‐497a‐5p targeted HECTD1 to regulate the neuroinflammation of LPS‐induced BV2 cells and the apoptosis of HT22 cells. (A) HECTD1 protein expression was measured by western blot (WB) analysis in BV2 cells transfected with si‐HECTD1 or si‐NC. (B, C) BV2 cells were transfected with anti‐miR‐NC + si‐NC, anti‐miR‐497a‐5p + si‐NC, or anti‐miR‐497a‐5p + si‐HECTD1 followed by being treated with or without LPS. (B) The protein levels of iNOS and COX2 were examined by WB analysis. (C) ELISA assay was used to determine the concentrations of IL‐6, IL‐1β, and TNF‐α. (D–G) The cell culture supernatant from LPS‐stimulated BV2 cells transfected with anti‐miR‐NC + si‐NC, anti‐miR‐497a‐5p + si‐NC, or anti‐miR‐497a‐5p + si‐HECTD1 was co‐cultured with HT22 cells for 12 h. CCK8 assay (D), EdU assay (E), and flow cytometry (F) were used to analyze cell viability, EdU^+^ cells, and apoptotic cells. (G) The protein levels of Bcl‐2, Bax, and cleaved caspase‐3 were detected by WB analysis. *p < .05; **p < .01; ***p < .001.

The rescue experiments were performed to explore whether miR‐497a‐5p regulated the neuroinflammation and neuron cells apoptosis by targeting HECTD1. After transfection with si‐HECTD1 into BV2 cells, we confirmed that HECTD1 protein expression was markedly decreased (Figure 6a). Then, BV2 cells were co‐transfected with anti‐miR‐497a‐5p and si‐HECTD1 followed by being treated with LPS. Function analysis showed that miR‐497a‐5p inhibitor reduced the protein expression levels of iNOS and COX2, as well as inhibited the concentrations of IL‐6, IL‐1β, and TNF‐α in LPS‐induced BV2 cells. However, the inhibitory effect of anti‐miR‐497a‐5p on neuroinflammation could be eliminated by HECTD1 knockdown (Figure 6b,c). The medium of transfected and treated BV2 cells was collected and then incubated with HT22 cells. Through CCK8 assay and EdU assay, we found that miR‐497a‐5p inhibitor could promote the viability and the EdU^+^ cells in HT22 cells, while HECTD1 knockdown could reverse these effects (Figure 6d,e). Meanwhile, miR‐497a‐5p inhibitor reduced the apoptotic cells, increased Bcl‐2 protein expression, and decreased the Bax and cleaved caspase‐3 protein expression. Also, silenced HECTD1 reversed the suppressive effect of miR‐497a‐5p inhibitor on the apoptosis of HT22 cells (Figure 6f,g). The above data confirmed that miR‐497a‐5p targeted HECTD1 to regulate the neuroinflammation of microglia cells and the apoptosis of neuron cells.

### 3.7 Circ‐Bnc2 targeted miR‐497a‐5p to upregulate HECTD1

**FIGURE 7:**
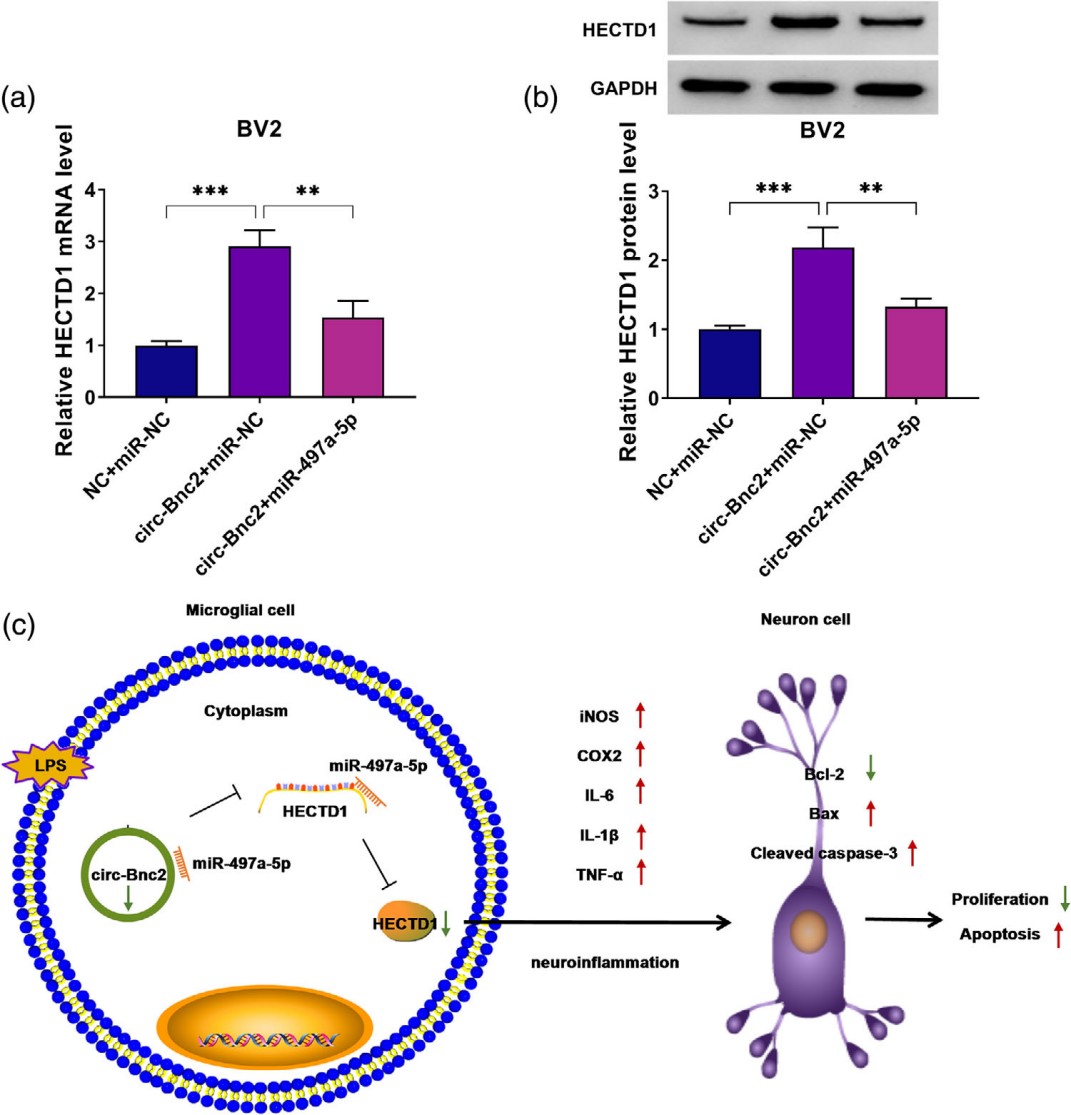
Circ‐Bnc2 targeted miR‐497a‐5p to upregulate HECTD1. (A, B) The mRNA and protein levels of HECTD1 were determined by qRT‐PCR and western blot (WB) analysis in BV2 cells transfected with NC + miR‐NC, circ‐Bnc2 + miR‐NC, or circ‐Bnc2 + miR‐497a‐5p. (C) The main idea diagram of this study. **p < .01; ***p < .001.

Our data showed that circ‐Bnc2 could sponge miR‐497a‐5p, and miR‐497a‐5p could target HECTD1. To confirm the regulation of circ‐Bnc2 on HECTD1, we measured the expression of HECTD1 in BV2 cells co‐transfected with circ‐Bnc2 overexpression vector and miR‐497a‐5p mimic. The results showed that circ‐Bnc2 overexpression could promote the mRNA and protein levels of HECTD1, while these effects could be reversed by miR‐497a‐5p overexpression (Figure 7a,b). To further evaluate, we assessed neuroinflammation and neuron cell apoptosis under the co‐transfection of circ‐Bnc2 and si‐HECTD1 or anti‐miR‐497a‐5p. Our data showed that HECTD1 knockdown reversed the inhibition of circ‐Bnc2 on the expression of iNOS and COX2, as well as the levels of IL‐6, IL‐1β, and TNF‐α in LPS‐induced BV2 cells (Figure S1a,b). Moreover, the promotion effect of circ‐Bnc2 on cell viability, EdU^+^ cell rate, and Bcl‐2 protein expression as well as the suppressive effect on Apoptotic cell rate, Bax protein expression, and cleaved caspase‐3 protein expression also could be abolished by HECTD1 knockdown (Figure S1c–f). The results showed that HECTD1 knockdown partially eliminated the circ‐Bnc2‐mediated inhibition on neuroinflammation and neuron cell apoptosis. On the contrary, anti‐miR‐497a‐5p could not effectively inhibit the inflammatory response of BV2 cells and reduce the apoptosis of HT22 cells mediated by circ‐Bnc2 (Figure S2a–f), indicating that the overexpression of circ‐Bnc2 had effectively adsorbed a large number of miR‐497a‐5p to protect neuron apoptosis. Therefore, our results indicated that circ‐Bnc2 could sponge miR‐497a‐5p to positively regulate HECTD1, thereby regulating the neuroinflammation of LPS‐induced microglial cells and the growth of neuron cells (Figure 7c).

## 4 DISCUSSION

The latest research has found that neuroinflammation is related to the occurrence of depression. Microglia are the first cells to respond to peripheral inflammatory signals, and their activation can initiate an inflammatory cascade to induce neuroinflammation (Jia et al., 2021; Singhal & Baune, 2017). Here, the role of circ‐Bnc2 in LPS‐stimulated microglia cells neuroinflammation and neuron cells apoptosis was explored to confirm its function in depression progression. Our data indicated that circ‐Bnc2 expression was decreased in LPS‐treated BV2 cells, and its upregulating could alleviate LPS‐induced neuroinflammation in BV2 cells to enhance HT22 cells proliferation and suppress apoptosis. These data confirm that circ‐Bnc2 had a neuroprotective effect, which might be a potential target for depression treatment.

Circ‐Bnc2 mainly localized in the cytoplasm indicated that circ‐Bnc2 might be involved in posttranscriptional regulation, which provided the necessary conditions for circ‐Bnc2 to become a sponge for miRNA. Through online software, we found that miR‐497a‐5p could be sponged by circ‐Bnc2. In the past research, miR‐497a‐5p was discovered to inhibit VEGF‐A protein expression to reduce retina neovascularization, thereby alleviating diabetic retinopathy progression (Zhang et al., 2017). miR‐497a‐5p was significantly overexpressed in mouse models of posttraumatic stress disorder and might be used to distinguish between different phenotypes of stress and susceptibility (Maurel et al., 2021). A new study showed that miR‐497a‐5p knockdown alleviated depression‐like behavior in rats and inhibited neuroinflammation by repressing the activation of microglia cells (Zhai et al., 2020). Consistent with this result, our data confirmed that miR‐497a‐5p inhibitor restrained LPS‐induced neuroinflammation to inhibit neuron cells apoptosis. In the rescue experiments, the inhibition effect of circ‐Bnc2 on neuroinflammation in microglia cells and apoptosis in neuron cells could be reversed by overexpressing miR‐497a‐5p, confirming that circ‐Bnc2 targeted miR‐497a‐5p to alleviate depression progression.

HECTD1 is an E3 ubiquitin ligase essential for neural tube closure and proper head mesenchymal development (Zohn et al., 2007). Previous studies suggested that the upregulation of HECTD1 could restore neuron cell function and alleviate blood–brain barrier injury (Bai et al., 2018). Tang et al. (2021) reported that HECTD1 could regulate astrocyte activation and had different effects under different concentrations of LPS. Zhang et al. (2020) showed that elevated expression of HECTD1 inhibited the microglial activation, thus alleviating the progression of depression. Our results pointed out that HECTD1 could be targeted by miR‐497a‐5p. Further experiments found that the absence of HECTD1 abolished the suppressive effect of anti‐miR‐497a‐5p on LPS‐induce neuroinflammation in microglia cells and apoptosis in neuron cells, suggesting that HECTD1 had a neuroprotective effect and HECTD1 was indeed a downstream target of miR‐497a‐5p. Additionally, the positive regulation effect of circ‐Bnc2 on HECTD1 improved the hypothesis of circ‐Bnc2/miR‐497a‐5p/HECTD1 axis in our research.

In summary, our results demonstrated that circ‐Bnc2 inhibited microglial activation by regulating miR‐497a‐5p/HECTD1 axis, thereby alleviating neuroinflammation and neuron cell apoptosis. Our results offer new insights into the pathogenesis of depression and provide a potential target for depression treatment.

## CONFLICT OF INTEREST STATEMENT

The authors declare no conflicts of interest.

### PEER REVIEW

The peer review history for this article is available at https://publons.com/publon/10.1002/brb3.2935.

## Supporting information

Supplementary Fig. 1 Effect of circ‐Bnc2 and si‐HECTD1 the neuroinflammation of LPS‐induced BV2 cells and the apoptosis of HT22 cells. (A‐B) BV2 cells were transfected with NC + si‐NC, circ‐Bnc2 + si‐NC or circ‐Bnc2 + si‐HECTD1 followed by treated with or without LPS. (A) WB analysis was performed to examine the protein levels of iNOS and COX2. (B) ELISA assay was utilized to assess the concentrations of IL‐6, IL‐1β and TNF‐α. (C‐F) The cell culture supernatant from LPS‐stimulated BV2 cells transfected with NC + si‐NC, circ‐Bnc2 + si‐NC or circ‐Bnc2 + si‐HECTD1 was co‐cultured with HT22 cells for 12 h. CCK8 assay (C), EdU assay (D) and flow cytometry (E) were performed to measure cell viability, EdU^+^ cells and apoptotic cells. (F) WB analysis was used to test the protein levels of Bcl‐2, Bax and cleaved caspase‐3. *P < 0.05, **P < 0.01, ***P < 0.001.

Supplementary Fig. 2 Effect of circ‐Bnc2 and anti‐miR‐497a‐5p the neuroinflammation of LPS‐induced BV2 cells and the apoptosis of HT22 cells. (A‐B) BV2 cells were transfected with NC + anti‐NC, circ‐Bnc2 + anti‐NC or circ‐Bnc2 + anti‐miR‐497a‐5p followed by treated with or without LPS. (A) WB analysis was used to test the protein levels of iNOS and COX2. (B) The concentrations of IL‐6, IL‐1β and TNF‐α were examined using ELISA assay. (C‐F) The cell culture supernatant from LPS‐stimulated BV2 cells transfected with NC + anti‐NC, circ‐Bnc2 + anti‐NC or circ‐Bnc2 + anti‐miR‐497a‐5p was co‐cultured with HT22 cells for 12 h. Cell viability, EdU^+^ cells and apoptotic cells were determined using CCK8 assay (C), EdU assay (D) and flow cytometry (E). (F) The protein levels of Bcl‐2, Bax and cleaved caspase‐3 were tested using WB analysis. *P < 0.05, **P < 0.01, ***P < 0.001.

## Data Availability

The datasets used and analyzed during the current study are available from the corresponding author on reasonable request.
