# Supplementary figures and images for: Circ‐Bnc2 alleviates neuroinflammation in LPS‐stimulated microglial cells to inhibit neuron cell apoptosis through regulating miR‐497a‐5p/HECTD1 axis
Source: Brain Behav. 2023 Mar 24;13(5):e2935. doi: 10.1002/brb3.2935 (PMC10175969; doi:10.1002/brb3.2935)

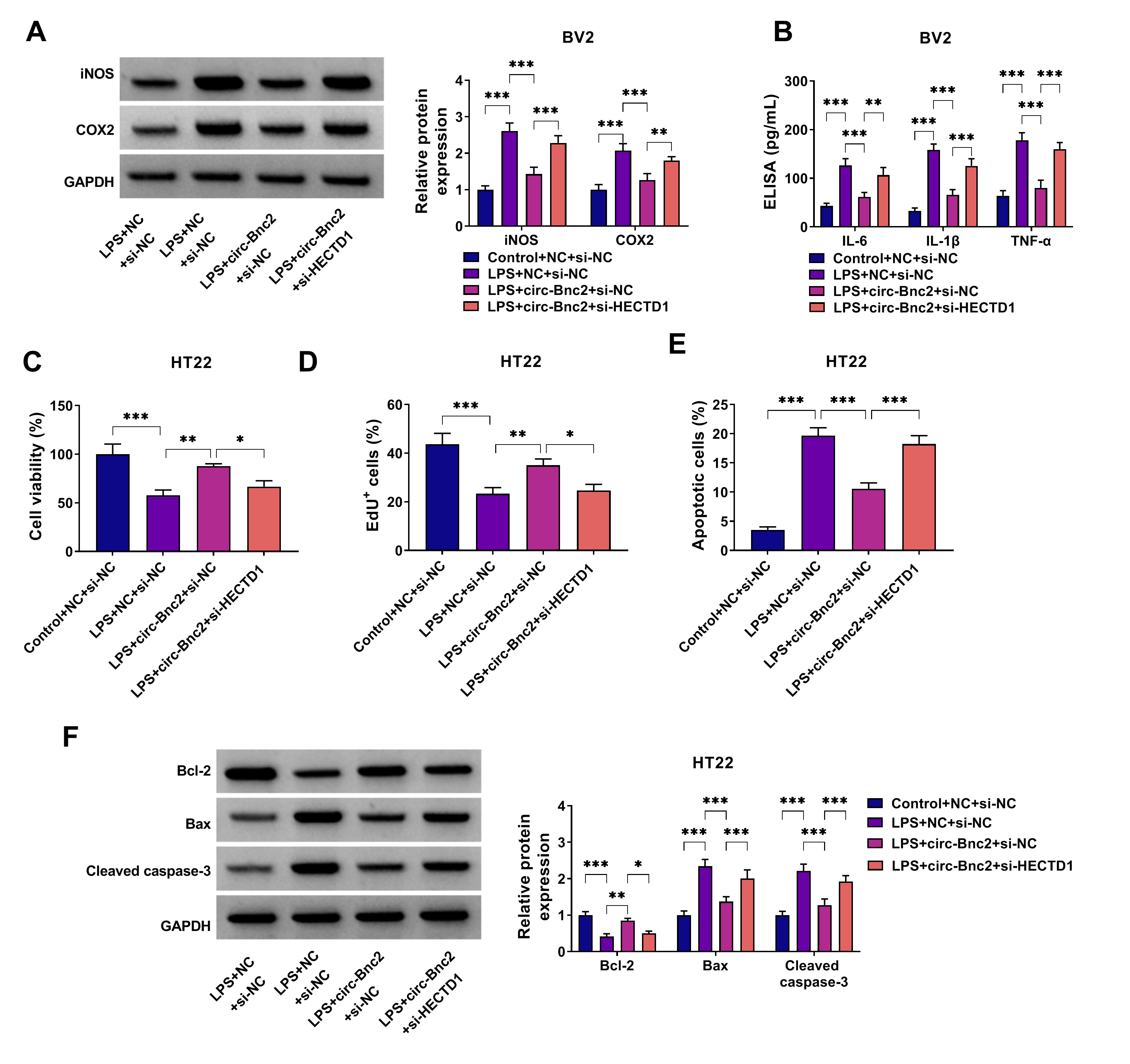

Supplement: Supplementary file 1 — Supplementary Fig. 1 Effect of circ‐Bnc2 and si‐HECTD1 the neuroinflammation of LPS‐induced BV2 cells and the apoptosis of HT22 cells. (A‐B) BV2 cells were transfected with NC + si‐NC, circ‐Bnc2 + si‐NC or circ‐Bnc2 + si‐HECTD1 followed by treated with or without LPS. (A) WB analysis was performed to examine the protein levels of iNOS and COX2. (B) ELISA assay was utilized to assess the concentrations of IL‐6, IL‐1β and TNF‐α. (C‐F) The cell culture supernatant from LPS‐stimulated BV2 cells transfected with NC + si‐NC, circ‐Bnc2 + si‐NC or circ‐Bnc2 + si‐HECTD1 was co‐cultured with HT22 cells for 12 h. CCK8 assay (C), EdU assay (D) and flow cytometry (E) were performed to measure cell viability, EdU+ cells and apoptotic cells. (F) WB analysis was used to test the protein levels of Bcl‐2, Bax and cleaved caspase‐3. *P < 0.05, **P < 0.01, ***P < 0.001. [file BRB3-13-e2935-s001.tif]

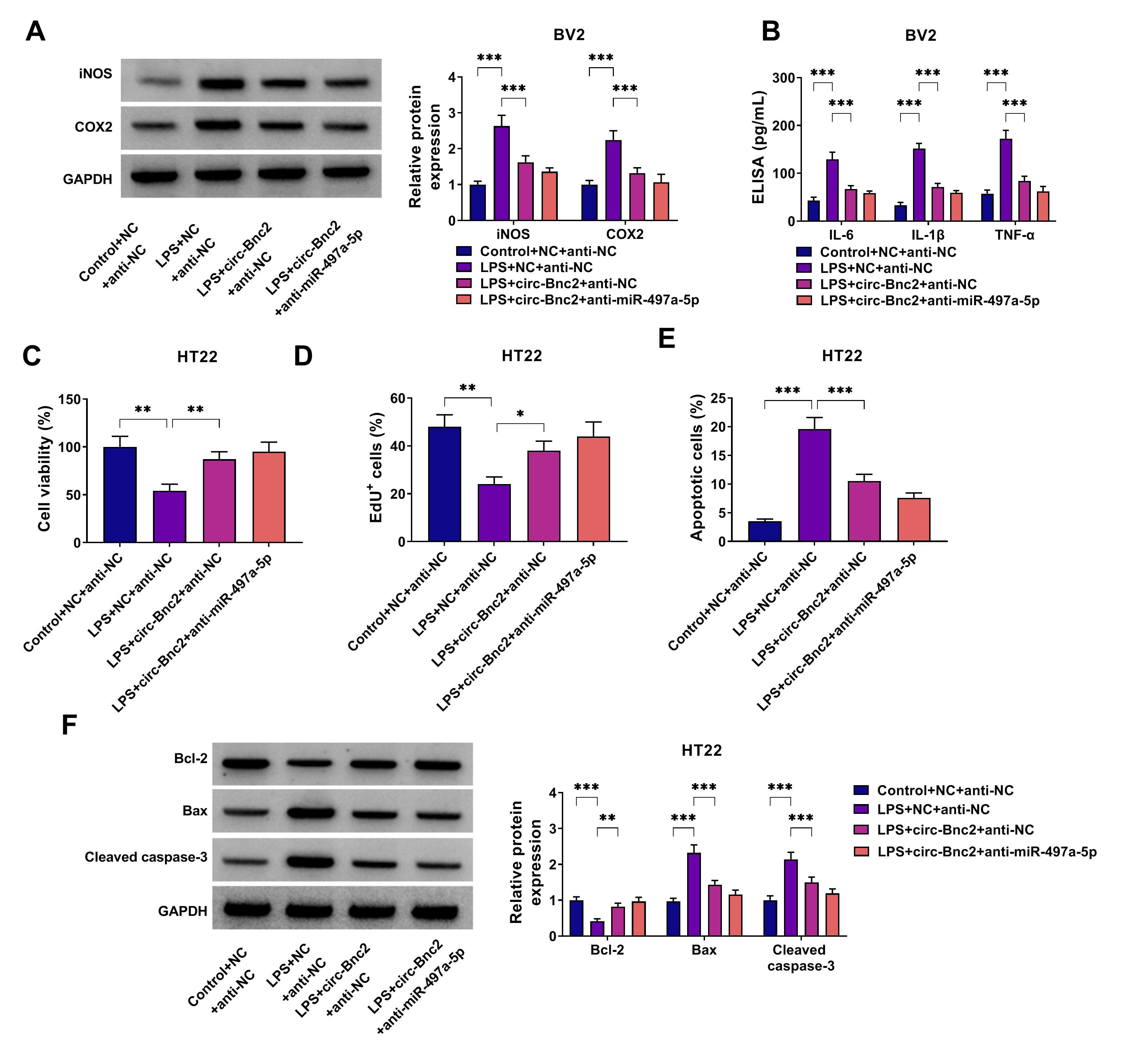

Supplement: Supplementary file 2 — Supplementary Fig. 2 Effect of circ‐Bnc2 and anti‐miR‐497a‐5p the neuroinflammation of LPS‐induced BV2 cells and the apoptosis of HT22 cells. (A‐B) BV2 cells were transfected with NC + anti‐NC, circ‐Bnc2 + anti‐NC or circ‐Bnc2 + anti‐miR‐497a‐5p followed by treated with or without LPS. (A) WB analysis was used to test the protein levels of iNOS and COX2. (B) The concentrations of IL‐6, IL‐1β and TNF‐α were examined using ELISA assay. (C‐F) The cell culture supernatant from LPS‐stimulated BV2 cells transfected with NC + anti‐NC, circ‐Bnc2 + anti‐NC or circ‐Bnc2 + anti‐miR‐497a‐5p was co‐cultured with HT22 cells for 12 h. Cell viability, EdU+ cells and apoptotic cells were determined using CCK8 assay (C), EdU assay (D) and flow cytometry (E). (F) The protein levels of Bcl‐2, Bax and cleaved caspase‐3 were tested using WB analysis. *P < 0.05, **P < 0.01, ***P < 0.001. [file BRB3-13-e2935-s002.tif]
